# An online community peer support intervention to promote COVID-19 vaccine information among essential workers: a randomized trial

**DOI:** 10.1080/07853890.2022.2138960

**Published:** 2022-10-31

**Authors:** Dominic Arjuna Ugarte, Jeremy Lin, Tianchen Qian, Sean D. Young

**Affiliations:** a Department of Emergency Medicine, University of California, Irvine, CA, USA; b Department of Statistics, University of California, Irvine, CA, USA; c Department of Informatics, University of California, Irvine, CA, USA

**Keywords:** Public health, vaccination hesitancy, help-seeking behaviour

## Abstract

**Introduction:**

Vaccine hesitancy is still rampant in the United States, including health care personnel. Vaccination of frontline essential workers (e.g. health care workers) is very important, especially during a pandemic. We tested the efficacy of a 4-week online, peer-led intervention (Harnessing Online Peer Education) to promote requests for COVID-19 vaccine information among essential workers.

**Methods:**

Participants (*N* = 120) and peer leaders (*N* = 12) were recruited through online advertisements from July 23 to August 20, 2021. Eligibility criteria included: 18 years or older, U.S. resident, English speaker, part of phase 1a or 1 b of COVID-19 vaccine rollout (e.g. frontline essential workers), hadn’t received a COVID-19 vaccine but able to receive one. This was a parallel assignment randomised trial. STATA was used to create a randomisation using a random number generator so that all possible assignments of participants and peer leaders to groups were equally likely. Participants were randomly assigned to intervention or control arms that consisted of two private, hidden Facebook groups, each with 30 participants. Peer leaders were randomly assigned to an intervention group, each with six peer leaders. Participants in the intervention arm were randomly assigned to three peer leaders. Participants were blinded after assignment. Peer leaders were tasked with reaching out to their assigned participants at least three times each week. Participants completed a baseline and a post intervention survey. The study is registered on ClinicalTrials.org under identifier NCT04376515 and is no longer recruiting. This work was supported by the NIAID under grant 5R01AI132030-05.

**Results:**

A total of 101 participants analysed (50 intervention and 51 control). Six people in the intervention group and 0 people in the control group requested vaccine information. Ten people in the intervention group and six people in the control group provided proof of vaccination. The odds of requesting vaccine information in the intervention group was 13 times that in the control group (95% confidence interval: (1.5, 1772), *p*-value = 0.015). Thirty-seven participants in the intervention group and 31 in the control group were engaged at some point during the study.

**Conclusions:**

Results suggest peer-led online community groups may help to disseminate health information, aid public health efforts, and combat vaccine hesitancy.

**Key Messages**
- The odds of requesting vaccine information was 13 times in the intervention group.
- Peer-led online communities may help to disseminate information and aid public health efforts to combat vaccine hesitancy.

## Introduction

Both initial and repeated vaccination of frontline essential workers (e.g. health care workers) is very important, especially during a pandemic [1,2]. However, vaccine hesitancy is still rampant in the US among the general population and essential workers [3,4], including health care personnel [5,6]. For example, among nursing home staff, booster rates are as low as 17% in some states [7].

The Harnessing Online Peer Education (HOPE) intervention has successfully led to attitude/behaviour change across multiple geographic regions and medical conditions [8,9] and might be applied to address COVID-19 vaccine hesitancy. HOPE utilises trained peer leaders to provide support to others through online groups [8,9]. To address the growing problems around vaccine hesitancy and need for vaccinations, we sought to pilot test the efficacy of the HOPE intervention to elicit an increase in requests for COVID-19 vaccine information among essential workers, and secondarily to explore potential effect sizes in vaccination differences to prepare for a future fully powered randomised trial on vaccinations.

## Methods

### Recruitment

Participants (*N* = 120) and peer leaders (*N* = 12) were recruited through online advertisements from July 23 to August 20, 2021. Sample size was based on expectation of behaviour change in online communities using sample sizes from previous HOPE studies [10]. In our previous HOPE study looking at HIV testing, sample size was set at 118 total, 59 per condition. We slightly over recruited to allow for loss to follow-up or drop out from the study. Ads were designed to target those living in the United States and who were frontline essential workers. When potential participants clicked on an ad, they were directed to a Qualtrics survey to check for eligibility. Eligibility criteria for participants were: 18 years or older, U.S. resident, English speaker, part of phase 1a or 1 b of COVID-19 vaccine rollout (e.g. health care workers, teachers, other frontline essential workers), hasn’t received a COVID-19 vaccine and does not have medical conditions preventing them from receiving one. Those who were not eligible were thanked for their interest in our study and told their information would not be used and destroyed for their confidentiality. Those who were eligible were called to confirm they were a unique person and to go over the study information sheet and obtain their verbal consent to participate in the study. 826 people (82.1%) who responded to our ads were either not eligible or not interested in being a participant. Hundred and seventy-nine people (17.8%) who responded to our ads were confirmed eligible to be a participant. Of those 179 people, 132 completed the baseline survey (73.7%). And of those 132 people, 120 people accepted the invite to the Facebook group (90.9%).

Peer leaders had similar eligibility criteria but had received at least the first dose of any COVID-19 vaccine and initially expressed vaccine hesitancy. To assess vaccine hesitancy, we asked peer leaders to describe whether they initially had any reasons to not take the vaccine and based response on reasons of discomfort and/or hesitancy. Peer leaders were also required to attend three online trainings, approximately 3 h each. 152 people (79.2%) who responded to our ads were either not eligible or not interested in being a peer leader. 40 people (20.8%) were confirmed eligible to be a peer leader and of those 40, 12 completed training (30.0%).

### Peer leader training

Training covered COVID-19 information/misinformation, the psychology of gaining trust through online communities, as well as study logistics and weekly topics. Session 1 focussed on COVID-19 information and COVID-19 vaccine misinformation. Session 2 focussed on stigma and politicisation of COVID-19, and briefly discussed motivational interviewing. Session 3 focussed on study logistics and weekly topics. For example, in week 1, we recommended not focussing on COVID-19 and just posting about friendly topics to build rapport. Overall, we let them know it was a free-flowing group and conversations within groups depended on how participants reacted and what participants posted. Throughout the sessions, peer leaders participated in group activities to practice posting and commenting on Facebook. This was done through a private, hidden Facebook group created just for the peer leaders. This group also remained open during the study as a resource for peer leaders to connect with the study team and each other. After each session, peer leaders were given homework (i.e. find and post a video about COVID-19 education) to help recap what they learned, practice engaging others online, and prepare them for the next session.

### Intervention

This was a parallel assignment randomised trial. STATA was used by our statistician to create a randomisation using a random number generator in a manner that all possible assignments of subjects to groups were equally likely. Similarly, all possible assignments of peer leaders to the intervention groups were equally likely. Participants were blinded after assignment. Enrolment and group assignment were handled by the study team. Participants were randomly assigned to intervention or control arms. Each arm consisted of two private, hidden Facebook groups, each with 30 participants. Peer leaders were randomly assigned to an intervention group, each with 6 peer leaders. Each participant in the intervention arm was randomly assigned to 3 peer leaders. Based on the clinicaltrial.gov’s definition of start date (The actual date on which the first participant was enrolled in a clinical study), the start date is actually August 19, 2021. While we did start recruiting on July 23, for our study, participants aren’t fully enrolled until they join the Facebook group. August 21 is when we had everyone in their respective groups and officially began the four-week Facebook portion. Participants completed a baseline and a post intervention survey online. Surveys collected demographic information, information about their internet use (including how much time they spend looking for information regarding COVID-19), general health information including if they’ve ever been tested for COVID-19 or received the COVID-19 vaccine and other recommended vaccinations, and their attitudes towards the COVID-19 vaccine (7-point Likert scale questions such as, ‘I am completely confident that the COVID-19 vaccine is safe,’ ‘I am completely confident that the COVID-19 vaccine will be effective in preventing COVID-19,’ and ‘I, or someone I know, has had a very negative experience after getting a vaccine, for example, strong side effects, or other medical issues.’). Participants, in both the control and intervention, were told the group was for them to use as they wanted and to continue using Facebook as they normally would. Participation and engagement in the online community group was voluntary, and participants could stop engaging/participating at any time. Participants could do everything a peer leader could do and could make their own posts, direct messages, and real-time chat with other participants and peer leaders. Peer leaders, however, were tasked with reaching out to their assigned participants at least three times each week. Those they reached out to and if there was any response was recorded each week on a response sheet. The study team met with the peer leaders individually once a week to check in, give feedback, and answer any questions. Participants were also reminded each week that they could request a brochure with more information about the COVID-19 vaccine, including vaccination sites. Participants were compensated for the online surveys that they completed: a $15 Amazon gift code for the baseline survey and $20 for the 4-week survey. Additionally, if participants stated on the follow-up survey that they had received the vaccine, they could receive another $15 Amazon gift code if they sent as a picture of their vaccine card for proof. Peer leaders were compensated a $20 Amazon gift code for each weekly response sheet they completed.

### Analysis

All analyses were conducted in R (version 4.1.2) We assessed the effect of the HOPE intervention on the primary outcome (whether a participant requested vaccine information during the study), and the secondary outcome (whether a participant got the vaccine during the study). We adjusted for race (dichotomised by Caucasian vs other), age (above vs. below 40), sex from birth, education (bachelor or below vs. grad school or above), and ethnicity (Latino vs. other).

The intervention effect on the primary outcome was assessed using Firth logistic regression instead of the usual logistic mixed effects models or generalised estimating equations to fix the separation issue (that 0 participants in the control group requested vaccine information). Due to the limitation of the Firth logistic regression software, we were unable to account for the clustering by Facebook group, which may lead to anti-conservative inference.

The intervention effect on the secondary outcome was assessed using generalised estimating equations with an exchangeable correlation structure ([2]), which accounts for the clustering by Facebook group.

Engagement in the study was defined as participants that posted, commented, voted, or reacted to anything within a given week.

### Ethics

This study was exempted by the University of California, Irvine Institutional Review Board. Research at UCI is eligible for exempt self-determination if it is not funded by the Department of Justice, does not involve special vulnerable populations, and falls under exempt category 1–3. Our research (category 3) was a behavioural information and ‘any disclosure of the human subjects’ responses outside the research would NOT reasonably place the subjects at risk of criminal or civil liability or be damaging to the subjects’ financial standing, employability, educational advancement, or reputation.’ (Please see the UCI Office of Research website: https://research.uci.edu/wp-content/uploads/confirmation-of-exempt-reference.pdf). The study is registered on ClinicalTrials.org under identifier NCT04376515. (CONSORT Checklist in supplementary files).

## Results

While 132 participants completed the baseline survey and were potentially randomised, 12 were lost to follow-up and either did not send us a friend request or did not join the group. A total of 120 participants were assigned to their respective groups. Twelve participants were later removed from analysis as it was discovered they had been vaccinated before the study began (6 in the intervention and six in the control) and another seven (4 in the intervention and 3 in the control) did not complete the follow-up survey. In total, 50/60 participants from the intervention arm and 51/60 participants from the control arm were analysed (CONSORT Flowchart in supplementary files). Mean age of the intervention and control groups was 39.9 ± 10.15 and 38.1 ± 11.63 respectively (Table 1). 79.6% (*N* = 43) of the intervention arm, and 77.8% (*N* = 42) of the control arm were female (Table 1). The majority of participants in both conditions (74.1%, control group; 88.9% intervention group) were in the healthcare field, followed by education, and other essential workers (Table 1).

**Table 1.** Descriptive Statistics of the Study Population.

	Category	Control Group	Intervention Group	Overall	*p* Value
*n*		54	54	108	
Age (mean (SD))		38.09 (11.63)	39.94 (10.15)	39.02 (10.90)	
Race (%)	White	42 (77.8)	40 (74.1)	82 (76.0)	0.664
	Black or African American	3 (5.6)	7 (13.0)	10 (9.3)	
	American Indian or Alaska Native	4 (7.4)	3 (5.6)	7 (6.5)	
	Asian	2 (3.7)	2 (3.7)	4 (3.7)	
	Other	2 (1.9)	1 (1.9)	3 (2.8)	
	Native Hawaiian or Pacific Islander	1 (1.9)	0 (0.0)	1 (0.9)	
	Not Reported	0 (0.0)	1 (1.9)	1 (0.9)	
Ethnicity (%)	Not Hispanic or Latino	39 (72.2)	45 (83.3)	84 (77.8)	0.201
	Hispanic or Latino	11 (20.4)	9 (16.7)	20 (18.5)	
	Not Reported	2 (3.7)	0 (0.0)	2 (1.9)	
	Unknown	2 (3.7)	0 (0.0)	2 (1.9)	
Sex at Birth (%)	Female	42 (77.8)	43 (79.6)	85 (78.7)	1
	Male	12 (22.2)	11 (20.4)	23 (21.3)	
Education (%)	High School Diploma or equivalent (GED)	3 (5.6)	0 (0.0)	3 (2.8)	0.005
	Some College/Certificate	6 (11.1)	5 (9.3)	11 (10.2)	
	Vocational/Trade School	0 (0.0)	7 (13.0)	7 (6.5)	
	Bachelor’s degree	23 (42.6)	22 (40.7)	45 (41.7)	
	Some Graduate or Professional School	10 (18.5)	2 (3.7)	12 (11.1)	
	Completed Graduate or Professional School	12 (22.2)	18 (33.3)	30 (27.8)	
Income (%)	$200,000 or more	3 (5.6)	4 (7.4)	7 (6.5)	0.144
	$150,000—$199,999	7 (13.0)	9 (16.7)	16 (14.8)	
	$100,000—$149,999	15 (27.8)	17 (31.5)	32 (29.6)	
	$75,000—$99,999	14 (25.9)	7 (13.0)	21 (19.4)	
	$50,000—$74,999	4 (7.4)	10 (18.5)	14 (13.0)	
	$35,000—$49,999	5 (9.3)	5 (9.3)	10 (9.3)	
	$25,000—$34,999	0 (0.0)	1 (1.9)	1 (0.9)	
	$10,000—$24,999	3 (5.6)	0 (0.0)	3 (2.8)	
	Less than $10,000	0 (0.0)	1 (1.9)	1 (0.9)	
	Prefer not to answer	3 (5.6)	0 (0.0)	3 (2.8)	
Political Ideology (%)	Republican	18 (33.3)	22 (40.7)	40 (37.0)	0.052
	Independent	22 (40.7)	11 (20.4)	33 (30.6)	
	Democrat	12 (22.2)	13 (24.1)	25 (23.1)	
	Other (please specify)	2 (3.7)	8 (14.8)	10 (9.3)	
Region (%)	South	18 (33.3)	13 (24.1)	31 (28.7)	0.291
	Midwest	15 (27.8)	10 (18.5)	25 (23.1)	
	West	14 (25.9)	21 (38.9)	35 (32.4)	
	Northeast	7 (13.0)	10 (18.5)	17 (15.7)	
Career (%)	Education	11 (20.4)	5 (9.3)	16 (14.8)	0.137
	Healthcare	40 (74.1)	48 (88.9)	88 (81.5)	
	Other Frontline Worker	3 (5.6)	1 (1.9)	4 (3.7)	

A total of six people in the intervention group and zero people in the control group requested vaccine information. The odds of requesting vaccine information in the intervention group was 13 times that in the control group (95% confidence interval: (1.5, 1772), *p*-value = 0.015) (Table 2). Note that the odds ratio would be positive infinity for information requests (as we have no one in control group who requested into). The Firth logistic regression is a reasonable approach to pull the odds ratio back to a finite number (in our case OR = 13), but due to the nature of the data and the exponential function, the upper CI is still very large. We found no group differences in the secondary outcome on odds of getting a vaccination (95% CI: (0.572, 5.871), *p* = 0.3) (Table 3). On the survey, 15 people reported receiving the vaccine (9 intervention and 6 control). One person in the intervention group was in follow-up with our team and got the vaccine after completing the survey. A total of 10 people in the intervention group and 6 people in the control group provided proof of vaccination.

**Table 2.** Primary outcome analysis – odds of requesting resources.

	Log odds ratio	95 % Confidence interval		*p* Value
		lower bound	upper bound	
(Intercept)	−5.71	−11.61	−2.46	<.001
Intervention	2.60	0.41	7.48	0.02
Race White	0.24	−1.52	2.58	0.80
Age Above 40 Years old	0.44	−1.18	2.23	0.59
Sex at Birth is Male	−0.03	−2.42	1.80	0.98
Education Graduate School or Professional School	−0.31	−2.15	1.31	0.71
Ethnicity not Latino	1.34	−1.10	6.29	0.32

**Table 3.** Secondary outcome analysis – odds of showing proof of vaccination.

		95 % Confidence interval		*p* Value
	Log odds ratio	lower bound	upper bound	
(Intercept)	−2.79	−5.69	0.12	0.06
Intervention	0.61	−0.558	1.77	0.31
Race White	−0.14	−1.47	1.19	0.83
Age Above 40 Years old	−0.66	−1.72	0.40	0.22
Sex at Birth is Male	−1.48	−3.50	0.55	0.15
Education Graduate School or Professional School	−0.29	−1.61	1.02	0.66
Ethnicity not Latino	1.77	−0.20	3.74	0.08

Peer leaders were assigned approximately 15 participants each to reach out to each week. Of the 12 peer leaders, half completed all their tasks and turned in a complete tracking sheet every week (2 in group 1 and 4 in group 2).

37 participants in the intervention group and 31 in the control group were engaged at some point during the study. In the intervention arm, 33 participants (55.0%) were ‘engaged’ during week 1, 29 (48.3%) during week 2, 11 (18.3%) during week 3, and 21 (35.0%) during week 4. In the control arm, 30 participants (50.0%) were engaged during week 1, 15 (25.0%) during week 2, 16 (26.7%) during week 3, and 7 (11.7%) during week 4. Overall, there were a total of 160 posts or comments in the intervention arm and 315 posts or comments in the control arm.

## Discussion

Results suggest that the HOPE intervention can increase requests for vaccine-related information, potentially helping to address vaccine hesitancy. While this study promoted COVID-19 vaccine information among essential workers, the intervention could be adapted to disseminate information to the general public and for other public health issues. This study has immediate public health implications as a potential tool to disseminate information during public health crises to combat misinformation. Health departments might use peer-led online communities, such as HOPE, to increase requests for vaccine-related information among essential workers. Although there were no significant differences in vaccination rates between conditions, this may be due to vaccination mandates that began during the study^11^, the pilot study not being powered to detect vaccination rate differences, and the small number of control participants who requested vaccine information. A future fully-powered randomised controlled trial is needed to assess vaccination rates.

In line with results from previous HOPE studies, [10] engagement decreased in both intervention and control arms overall but engagement remained higher in the intervention arm through most of the study. This further supports the use of peer leaders to deliver health information.

Limitations include small sample size and short study duration. Previous studies using HOPE lasted 12 weeks [8,9]. The longer duration might further improve group differences. Also, not all peer leaders had completed their tasks every week. Some peer leaders had been affected by stress from the pandemic and being part of the healthcare field had at times been overworked during this time period. Although, not everyone completed their assigned tasks (6 out of 12 peer leaders), that also did not necessarily mean that they were not participating in the group. They still attempted to react and comment on posts they saw but may not have reached out to all their participants or not reached the quota of three attempts per week. If more peer leaders had been able to complete all their tasks each week, the effect differences between groups may have been more significant. While there was a significant difference in education between groups, we adjusted for this in the analysis to reduce any potential selection bias. For education, the significant p-value was driven by the cells with zero or very small counts (e.g. for education, the categories ‘High School Diploma or Equivalent (GED)’ and ‘Vocational/Trade School’. When we dichotomised education by ‘Bachelor or Under’ vs. ‘Graduate School or Above’ (as is done in the analysis), there is no significant difference between the two groups *(p*-value = .693). Therefore, the imbalance in education is unlikely to cause bias in the estimated intervention effect.

Overall, results suggest that peer-led online groups can be a powerful tool to disseminate health information, aid public health efforts, and may help to combat vaccine hesitation.

## Author contributions

**Dominic Arjuna Ugarte:** Conceptualisation, Validation, Investigation, Data Curation, Writing – Original Draft, Writing – Review and Editing, Project Administration. **Jeremy Lin:** Formal analysis, Visualisation. **Tianchen Qian:** Formal Analysis, Writing – Review and Editing. **Sean D. Young:** Conceptualisation, Methodology, Resources, Writing – Review and Editing, Supervision, Project Administration, Funding Acquisition.

All authors were involved in the final approval of this manuscript version and agree to be accountable for all aspects of the work.

## Data Availability

Deidentified data available on request due to privacy/ethical restrictions.
